# Prestorage Leukoreduction of Canine Blood Products: A Critically Appraised Topic

**DOI:** 10.3390/vetsci13090865

**Published:** 2026-08-26

**Authors:** Daniela Proverbio, Roberta Perego, Eva Spada

**Affiliations:** Dipartimento di Medicina Veterinaria e Scienze Animali (DIVAS), Università degli Studi di Milano, 26900 Lodi, Italy; roberta.perego@unimi.it (R.P.); eva.spada@unimi.it (E.S.)

**Keywords:** leukoreduction, canine transfusion medicine, blood products, storage lesions, transfusion reactions

## Abstract

Blood transfusion is a life-saving intervention in canine critical care, but the clinical value of prestorage leukoreduction remains uncertain. This Critically Appraised Topic addressed whether leukoreduced canine blood products, compared with corresponding non-leukoreduced products, improve product quality, reduce recipient inflammatory responses or transfusion reactions, and improve patient-centered outcomes. PubMed, Scopus, Web of Science, and CAB Abstracts were searched from inception to 10 March 2026 using a common search strategy. Twenty-eight comparative articles were retained for appraisal after exclusion of five reports that did not contain a direct leukoreduced-versus-non-leukoreduced comparison aligned with the structured question. Most of the available evidence concerns laboratory and storage outcomes. Leukoreduction modified selected biochemical, inflammatory, oxidative, and cellular markers, but the direction and consistency of the findings varied across outcomes and studies. Clinical studies did not demonstrate a consistent reduction in transfusion reactions or improvement in survival or recovery. Current evidence therefore supports leukoreduction primarily as a blood-product processing strategy; it is insufficient to establish a patient-centered benefit from routine universal leukoreduction in dogs.

## Simple Summary

Blood transfusion is an important and often life-saving treatment in dogs affected by severe anemia, bleeding disorders, or critical illness. During storage, blood products may undergo biological changes—known as storage lesions—that can influence their quality. White blood cells present in stored blood may contribute to these changes by releasing inflammatory mediators. Leukoreduction removes white blood cells before storage and is widely used in human transfusion medicine, but its routine use in veterinary medicine remains debated. This critically appraised topic evaluated the available evidence comparing leukoreduced and non-leukoreduced canine blood products. Twenty-eight comparative articles were included after exclusion of five reports that did not contain a direct LR–NLR comparison aligned with the structured question. Most product-level studies indicate that prestorage leukoreduction modifies or attenuates selected biochemical, cellular, metabolic, and inflammatory changes during storage, although the consistency and clinical importance of these effects vary by outcome and study design. Current clinical evidence is insufficient to determine whether routine universal leukoreduction reduces transfusion reactions or improves patient-centered outcomes in dogs.

## 1. Introduction

Blood transfusion is a life-saving intervention in canine medicine and represents an essential component of modern veterinary critical care. It is frequently used in dogs for the management of anemia, hemorrhage, coagulopathies, and various life-threatening conditions. The development of veterinary blood banking systems has led to improved availability of canine blood components, including whole blood (WB), packed red blood cells (pRBCs), and plasma products. As the use of transfusion therapy increases, greater attention has been directed toward optimizing blood product quality and minimizing potential transfusion-related complications [1,2].

During storage, blood components undergo biochemical, metabolic, and structural alterations collectively known as storage lesions. Residual leukocytes may undergo activation or degradation and release cytokines, proteolytic enzymes, reactive oxygen species, and other biologically active mediators with possible consequences the efficacy and safety of transfused products. Leukoreduction is performed to reduce the number of donor leukocytes present in transfused blood components and may consequently modify storage-related changes and the accumulation of inflammatory mediators. Leukoreduction may be achieved by removal of the buffy coat, component preparation by apheresis, or, more effectively, by leukocyte-reduction filtration. Filtration can be applied to whole blood before component separation, to individual red-cell or platelet components before storage, or at the bedside immediately before transfusion. Prestorage filtration is generally preferred because leukocytes are removed before they deteriorate and release mediators into the stored product, providing a more standardized reduction in residual leukocyte content. Filter material, filtration timing, temperature, blood component, and residual leukocyte count may influence the final characteristics of the leukoreduced product. In human transfusion medicine, leukoreduction is principally intended to reduce febrile non-hemolytic transfusion reactions, HLA alloimmunization and platelet refractoriness, and the transmission of leukocyte-associated infectious agents [3].

In veterinary medicine, routine leukoreduction remains inconsistent and its clinical benefits in dogs are uncertain. Experimental studies suggest favorable effects on selected indicators of blood-product quality, whereas clinical investigations of transfusion reactions and patient-centered outcomes have produced heterogeneous findings. Accordingly, the aim of this Critically Appraised Topic (CAT) was to summarize and critically evaluate the available evidence comparing leukoreduced and non-leukoreduced canine blood products.

## 2. Clinical Scenario

A 10-year-old, 28-kg mixed-breed dog is presented to a veterinary referral hospital with acute weakness, pale mucous membranes, tachycardia, and abdominal distension. Abdominal ultrasonography identifies a ruptured splenic mass with hemoabdomen, and an emergency splenectomy is performed. During the postoperative period, the dog remains tachycardic and poorly perfused, with a packed cell volume of 17% and clinical signs of inadequate oxygen delivery. Mild diffuse surgical-site oozing is also observed, and coagulation testing reveals prolonged prothrombin and activated partial thromboplastin times. Red-cell support is indicated for the clinically relevant anemia, while plasma may also be required to replace coagulation factors if active bleeding associated with coagulopathy persists. The hospital blood bank has both leukoreduced and non-leukoreduced products available. The clinician must decide whether to administer leukoreduced or non-leukoreduced packed red blood cells and, if plasma transfusion is indicated, whether the evidence supports selecting plasma derived from leukoreduced whole blood. Because the dog is critically ill, has recently undergone major surgery, and may require more than one transfusion, the clinician wishes to know whether prestorage leukoreduction meaningfully preserves blood-product quality, reduces inflammatory or transfusion reactions, or improves patient-centered clinical outcomes compared with non-leukoreduced products.

## 3. Structured Question

In dogs requiring transfusion, does the administration of prestorage leukoreduced blood products, compared with corresponding non-leukoreduced products, improve blood-product quality, reduce recipient inflammatory responses or transfusion reactions, and improve patient-centered clinical outcomes?

### PICO

- Population: Dogs requiring transfusion and canine blood products intended for transfusion.
- Intervention: Prestorage leukoreduced whole blood, packed red blood cells, or plasma derived from leukoreduced blood.
- Comparison: Corresponding non-leukoreduced blood products.
- Outcomes: Blood-product quality, recipient biological responses, transfusion reactions, survival, recovery, and other patient-centered outcomes.

## 4. Search Strategy

PubMed, Scopus, Web of Science, and CAB Abstracts were searched from database inception to 10 March 2026. The following common strategy was applied, with only the syntax necessary to run it in each database adapted: (leukoreduction OR leukodepletion OR “leukocyte reduction”) AND (“whole blood” OR “packed red blood cells” OR erythrocytes OR plasma OR “fresh frozen plasma”) AND (transfusion OR storage OR cytokine OR coagulation OR outcome OR survival) AND (dog OR canine OR veterinary). Reference lists of relevant articles were also checked. Searches were conducted in title, abstract, and keyword or indexing-term fields where supported by the database interface; otherwise, the closest available equivalent topic field was used.

### 4.1. Eligibility Criteria

Original studies involving dogs or canine blood components were considered eligible when they evaluated whole blood, packed red blood cells, erythrocyte concentrates, or plasma products; clearly identified the leukoreduction procedure; directly compared a leukoreduced product with the corresponding non-leukoreduced product; and reported at least one relevant product-level, recipient-biological, transfusion-reaction, hemostatic, or patient-centered outcome. Studies without a direct LR–NLR comparison, pre/post-filtration studies without an unfiltered control, studies comparing only different leukoreduced products, and technical assessments of filter performance without an NLR comparator were excluded. Reviews, editorials, case reports, case series, conference abstracts, and non-canine studies without separately extractable canine data were also excluded. Operational or methodological studies were eligible only when they included a direct LR–NLR comparison relevant to blood-component processing or quality.

### 4.2. Study Classification and Data Extraction

Each article was assigned to one principal study category:

- Laboratory studies evaluated blood units, subunits, aliquots, or samples without transfusion to a recipient.
- Experimental transfusion studies evaluated recipient responses under controlled conditions, frequently using healthy dogs or autologous transfusion models.
- Clinical studies involved dogs receiving transfusions for a clinical indication and were further described according to their specific design, including randomized controlled trials, prospective observational studies, retrospective cohort studies, and pilot clinical trials.
- Operational/methodological studies evaluated practical aspects of blood-component preparation and were retained only when an appropriate LR–NLR comparison was present.

For each article, the following information was extracted when available: study design; number of donors, recipients, donations, units, subunits or transfusions; blood component; leukoreduction method and timing; filter type; residual leukocyte count or percentage leukocyte removal; storage duration and conditions; comparator; outcomes; principal quantitative findings; statistical significance; and clinically relevant limitations. Dogs, blood units, aliquots, and transfusion episodes were reported separately to avoid conflating biological subjects with experimental units. Information not reported in the source was indicated as NR. Full-text eligibility assessment was conducted by one author, whereas data extraction and independent verification were performed by two authors. One report assessed during the appraisal had been co-authored by members of the present author group: Spada et al. (2021) [4]. To limit potential bias, the same predefined eligibility, extraction, and appraisal criteria were applied to all reports irrespective of authorship, and the reasons for inclusion, exclusion, and study-level ratings are reported explicitly.

### 4.3. Outcome Classification

Outcomes were classified into five predefined domains.

- Blood-product outcomes included biochemical, metabolic, cellular, inflammatory, morphological, oxidative, microbiological, and coagulation-related measurements obtained directly from stored blood components.
- Recipient biological responses included post-transfusion inflammatory, hematological, or immunological biomarkers measured in transfused dogs.
- Transfusion-reaction outcomes included overall acute transfusion reactions, febrile non-hemolytic transfusion reactions, and other specifically reported transfusion complications.
- Patient-centered outcomes included survival, survival to discharge, in-hospital mortality, length of hospitalization, clinical recovery, and organ dysfunction.
- Plasma and hemostatic outcomes included coagulation times, individual coagulation-factor activities, fibrinogen, von Willebrand factor, and other measures of hemostatic function.
- Product-level measurements and recipient biomarkers were considered surrogate or intermediate outcomes and were not interpreted as direct evidence of clinical benefit. Findings concerning whole blood, packed red blood cells, and plasma were appraised separately because these components differ in processing, residual leukocyte content, storage characteristics, and clinical purpose.

## 5. Search Outcome

The search identified 280 records (Scopus, 56; PubMed, 48; Web of Science, 135; CAB Abstracts, 41). After 159 duplicates were removed using Mendeley, 121 titles and abstracts were independently screened by D.P. and E.S.; disagreements were resolved through discussion. Eighty-eight records were excluded. Full texts were assessed for eligibility by D.P. Data were extracted by D.P. and verified by E.S., with discrepancies resolved through discussion. Twenty-eight articles were retained for critical appraisal (Table 1). 

## 6. Appraisal of the Evidence

Evidence was critically appraised with particular attention to the appropriateness of the LR–NLR comparator, sample size and experimental unit, randomization and blinding when relevant, control of confounding, clarity of the leukoreduction procedure, completeness of outcome reporting, statistical analysis, directness of the outcome, and clinical applicability. Formal numerical levels of evidence and the former good/fair/poor ratings were not retained because laboratory, experimental-recipient, and clinical designs could not be meaningfully ranked using a single numerical scale. An author-defined framework was used to classify the directness of the evidence according to its proximity to the clinical question. Category A included controlled clinical studies in dogs transfused for a clinical indication; Category B included observational clinical studies; Category C included experimental recipient studies; and Category D included product-level laboratory, storage, or operational studies. These categories describe evidence directness and should not be interpreted as a validated level-of-evidence hierarchy or as a measure of methodological quality. Methodological robustness was assessed independently of evidence directness using the predefined criteria reported in Table 2. Studies were classified as having Higher, Moderate, or Lower robustness according to the appropriateness of the LR–NLR comparator, study design and allocation methods, randomization and blinding where applicable, sample adequacy, completeness of procedural and outcome reporting, appropriateness of the statistical analysis, and control of relevant confounding factors. Robustness was assigned independently of the direction or statistical significance of the findings. The study-level application of the evidence categories and methodological robustness ratings is reported in Table 3.

## 7. Results

The final evidence set comprised 28 comparative articles: three Category A controlled clinical studies [13,14,18], three Category B observational clinical studies [15,19,26], two Category C experimental recipient studies [16,17], and 20 Category D product-level studies [1,2,4,5,6,7,8,9,10,11,12,20,21,22,23,24,25,27,28,29]. Methodological robustness was rated as Higher for one study, Moderate for 25 studies, and Lower for two studies. The literature was therefore dominated by laboratory and storage investigations evaluating whole blood, packed red blood cells, erythrocyte concentrates, or plasma products. Direct clinical evidence was comparatively limited. Only a small number of studies evaluated plasma or hemostatic outcomes [4,6,11,27]. Study designs, sample units, blood components, leukoreduction procedures, and principal LR–NLR findings are summarized in Table 1; the criteria and study-level application of the evidence classifications are reported in Table 2 and Table 3.

## 8. Discussion

The central finding of this CAT is the distinction between product-level effects and demonstrated benefits to the recipient. Product-level studies indicate that prestorage leukoreduction modifies or attenuates selected biochemical, cellular, metabolic, and inflammatory changes during storage. However, the direction, magnitude, and consistency of these effects vary according to the blood component, filtration procedure, storage duration, and outcome evaluated. Therefore, a statistically significant difference in a storage-related marker should not automatically be interpreted as a clinically meaningful improvement. Evidence from transfused dogs is considerably more limited and heterogeneous. Transfusion reactions and survival are influenced by the recipient’s underlying disease, blood-component age and dose, administration and monitoring practices, and immunohematological compatibility, in addition to residual leukocyte content. Incompatibilities involving canine erythrocyte antigens not routinely assessed in standard donor typing, including DEA 2, DEA 3, and Dal, may also contribute to transfusion-reaction risk [30]. Leukoreduction should therefore not be considered the sole determinant of transfusion safety. The available evidence does not demonstrate that routine leukoreduction consistently reduces transfusion reactions or improves survival or other patient-centered outcomes. However, this absence of demonstrated benefit should not be interpreted as evidence that leukoreduction is ineffective in every clinical setting. Potential benefits in selected populations, such as repeatedly transfused or markedly inflammatory patients, have not been adequately investigated.

### 8.1. Blood-Product Quality

Most included articles evaluated laboratory or storage outcomes. Across whole-blood and pRBC studies, leukoreduction was associated with lower values for selected markers of leukocyte-derived inflammation, hemolysis, oxidative injury, neutrophil extracellular traps, microparticles, lactate, or potassium, and with preservation of selected metabolic characteristics [1,2,6,7,10,11,20,21,22,23,24,25,29]. These effects were outcome- and time-dependent and were not reproduced uniformly across studies [1,5,9,20,29]. Filtration could also cause cellular or product losses, including platelet removal or changes in final product weight [1,11,12]. The evidence therefore indicates modification of selected storage-related characteristics, rather than a uniform improvement in every measure of blood-product quality.

### 8.2. Recipient Biological Responses

A small number of experimental recipient and controlled clinical studies measured inflammatory or immunological responses after transfusion [13,14,16,17]. Some reported differences in selected post-transfusion markers [13,17], whereas others found no LR–NLR differences [14,16]. These biomarkers represent intermediate outcomes and cannot, by themselves, establish a patient-centered clinical benefit.

### 8.3. Transfusion Reactions and Patient-Centered Outcomes

Clinical evidence was limited and inconsistent. One retrospective study reported a lower incidence of febrile non-hemolytic transfusion reactions with leukoreduced pRBCs but found no reduction in other acute complications or in-hospital mortality [15]. Randomized clinical trials and larger observational studies did not demonstrate a consistent reduction in overall acute transfusion reactions [13,14,18,19,26]. Available studies also did not establish an improvement in survival to discharge, duration of hospitalization, or other major patient-centered outcomes [13,14,15,18,19]. Small samples, low event rates, patient heterogeneity, nonrandomized product allocation in observational studies, and variable outcome definitions limited precision; however, the absence of demonstrated benefit cannot be attributed to inadequate statistical power alone.

### 8.4. Plasma Products

Plasma evidence was interpreted separately from evidence concerning cellular products. Comparative product-level studies evaluated plasma obtained from leukoreduced and non-leukoreduced whole blood [4,6,11,27]. Overall coagulation activity was largely preserved, and one crossover study detected no significant LR–NLR differences in the evaluated hemostatic variables [27]. Another controlled study reported prolonged activated partial thromboplastin time and reduced factor XI activity in LR plasma, while the other evaluated hemostatic variables remained unaffected [4]. These findings were derived from laboratory measurements, and no study demonstrated that plasma obtained from leukoreduced blood provided a clinical hemostatic advantage in bleeding dogs [4,27].

### 8.5. Clinical Implications

Applied to the clinical scenario, leukoreduced pRBCs may be considered when available as a blood-product processing option. However, current evidence does not justify assuring clinicians or owners that their use will reduce transfusion-reaction risk or improve survival. Plasma should be administered only for an appropriate hemostatic indication, and selection of plasma derived from leukoreduced blood cannot currently be justified on the basis of a demonstrated clinical hemostatic advantage. Decisions regarding leukoreduction should also consider product availability, processing-related cellular or volume losses, cost, and local blood-bank procedures.

### 8.6. Limitations

This CAT was limited by the restriction to English-language publications, exclusion of grey literature and conference abstracts, full-text eligibility assessment by one reviewer, and the absence of a quantitative synthesis. Title-and-abstract screening was performed independently by two reviewers, whereas data extraction was conducted by two authors, with disagreements resolved through discussion. These procedures reduced, but did not eliminate, the risk of selection and extraction errors. The evidence base was dominated by small product-level laboratory studies and included few randomized clinical trials. Additional limitations included heterogeneous blood components and leukoreduction procedures, variable storage periods, non-uniform outcome definitions, small clinical samples, and low transfusion-reaction event rates. Finally, evidence directness and methodological robustness were assessed using an author-defined framework rather than a formally validated level-of-evidence or risk-of-bias instrument. Some members of the author group had co-authored one included study and one full-text report that was subsequently excluded; although predefined criteria were applied uniformly and all decisions were reported transparently, a potential for author-related appraisal bias cannot be completely excluded.

## 9. Clinical Bottom Line

Most product-level studies indicate that prestorage leukoreduction modifies or attenuates selected biochemical, cellular, metabolic, and inflammatory changes during storage of canine blood products. The magnitude, direction, and consistency of these effects vary according to the blood component, outcome, storage duration, and study design. Current clinical evidence does not demonstrate that routine leukoreduction consistently reduces transfusion reactions or improves survival, recovery, or other patient-centered outcomes in dogs.

## 10. Conclusions

Prestorage leukoreduction modifies selected characteristics of stored canine blood products and may attenuate some storage-related changes. However, a consistent clinical advantage has not been demonstrated. Its routine use should therefore be described as a blood-product processing strategy rather than as an intervention proven to reduce transfusion reactions or improve patient-centered outcomes.

## Figures and Tables

**Table 1:** Characteristics, leukoreduction/blood-processing methods, and principal findings of comparative studies evaluating leukoreduced (LR) and non-leukoreduced (NLR) canine blood products.

#	Study	Study Design	LR/Blood-Processing Method	Sample	Product and Outcome	Main LR-NLR Finding
1	Antognoni 2021 [1]	Laboratory; paired split-unit controlled storage study	WB divided into paired subunits; one was filtered before storage and the other remained unfiltered.	7 dogs/14 WB subunits	WB; CBC, LDH, electrolytes, morphology, hemolysis; 42 d	LR: lower LDH and morphological index; most units reached 1% hemolysis at day 42
2	Corsi 2014 [2]	Laboratory; randomized controlled storage study	Erythrocyte concentrates assigned to prestorage filtration or unfiltered storage.	10 dogs/10 EC units	Erythrocyte concentrates; IL-1β, IL-8, TNF-α, IL-10; 35 d	IL-8 higher in NLR on days 28 and 35; no group effect for IL-1β, IL-10, or TNF-α
3	Frank 2021 [5]	Laboratory; two-period crossover storage study	WB donations underwent prestorage filtration or remained unfiltered according to the crossover period.	8 dogs/16 WB donations	WB; N-methylhistamine; 28 d plus simulated transfusion	NMH increased immediately after LR but did not differ between LR and NLR during 28-d storage
4	Graf 2012 [6]	Laboratory; controlled storage study	WB was filtered before separation and storage of pRBC and FFP; controls were prepared from unfiltered WB.	10 donors/10 WB units; FFP subset: 2 LR and 2 NLR units.	pRBC and FFP; VEGF; 21 d	VEGF remained <9 pg/mL in LR pRBC/FFP; NLR pRBC medians 37, 164, and 110 pg/mL on days 7, 14, and 21
5	Herring 2013 [7]	Laboratory; randomized controlled storage study	RBC concentrates assigned to prestorage filtration or unfiltered storage.	11 dogs/11 RBC units	RBC concentrates; phosphatidylserine-expressing microparticles; 35 d	Microparticles higher in NLR on days 14–35; 35-d increase 1.8-fold LR vs 5.5-fold NLR
6	Lucchese 2020 [8]	Laboratory; paired artificial-infection storage model	Artificially infected WB units were split into filtered LR and unfiltered NLR arms before storage.	5 WB units split LR/NLR	WB; Rickettsia conorii viability; 35 d	LR-WB required a longer time to achieve culture positivity, suggesting a lower bacterial burden; however, viable R. conorii persisted until day 35 in 3/5 WB and 3/5 LR-WB units. Leukoreduction reduced but did not eliminate the organism.
7	Muro 2017 [9]	Laboratory; crossover storage/simulated-transfusion study	pRBC units prepared with prestorage filtration or without filtration.	8 dogs/16 WB donations; each pRBC unit divided into two storage subunits	pRBC; phosphatidylserine and eicosanoids; 10/21 d	No LR effect on PS; transient post-filtration TXB2/PGF2α rise; no LR reduction of 6-keto-PGF1α accumulation
8	Smith 2015 [10]	Laboratory; randomized controlled storage study	Erythrocyte concentrates assigned to prestorage filtration or NLR storage.	10 dogs/10 EC units	Erythrocyte concentrates; procoagulant phospholipid; 35 d	PPL increased during storage; LR reduced the increase in procoagulant activity over time
9	Stefani 2021 [11]	Laboratory; paired split-unit storage study	WB or pRBC bags were divided into paired filtered and unfiltered subunits before storage; plasma was obtained during component preparation.	10 donors; 5 WB and 5 pRBC bags split LR/NLR	WB, pRBC and FFP; metabolic, cytokine and coagulation outcomes	At day 35 LR had lower lactate and higher pH; LR pRBC had lower K; IL-8 increased in NLR units
10	Trinder 2022 [12]	Operational; randomized comparative processing study	WB collections were randomized to prestorage filtration or standard unfiltered component processing.	68 WB collections (34/group)	pRBC/plasma weights and processing time	No group difference in final pRBC/plasma weights or processing time; LR pRBC lost a greater weight proportion (*p* < 0.01)
11	Bosch Lozano 2019 [13]	Clinical; prospective randomized blinded pilot trial	Clinically administered pRBC units were prepared with prestorage filtration or as unfiltered controls.	23 dogs (11 LR/12 NLR)	pRBC ≤12 d; inflammation, reactions, survival	Only 24-h WBC differed; reactions 1/11 LR vs 2/12 NLR; survival 8/11 vs 9/12
12	Claus 2022 [14]	Clinical; randomized blinded controlled preliminary trial	Blood-bag processing was randomized to prestorage filtration or NLR preparation before clinical transfusion.	61 dogs (34 LR/27 NLR)	RBC; leukocytes, IL-6, IL-8, MCP-1, CRP	No group differences for any biomarker over time (all *p* ≥ 0.18); in-hospital euthanasia 32% LR vs 15% NLR (*p* = 0.14)
13	Davidow 2022 [15]	Clinical; retrospective two-center study	Clinical pRBC transfusions were classified according to prestorage LR or NLR processing recorded by the participating centers.	455 dogs/730 transfusions	pRBC; acute complications and mortality	FNHTR 1% LR vs 4.5% NLR (*p* = 0.03); no reduction in other complications or in-hospital mortality
14	Callan 2013 [16]	Experimental; prospective randomized mixed parallel/crossover autologous transfusion study	Autologous pRBC units were prepared with or without prestorage filtration and stored for 7 or 28 days.	24 dogs overall; 20 transfused dogs/40 autologous pRBC transfusions (10 LR and 10 NLR dogs); 8 saline-control infusions	pRBC stored 7 vs 28 d; circulating inflammatory analytes	No LR–NLR differences were detected in the measured circulating analytes; 28-day pRBC transfusions induced an inflammatory response irrespective of leukoreduction or infusion rate.
15	McMichael 2010 [17]	Experimental; controlled autologous transfusion study	Autologous pRBC units were prepared with prestorage filtration or without filtration and stored for 21 days.	13 dogs (6 NLR/5 LR analyzed)	pRBC stored 21 d; post-transfusion inflammatory markers	WBC, neutrophils, fibrinogen and CRP rose after NLR transfusion (all *p* < 0.001) but not after LR transfusion
16	Radulescu 2021 [18]	Clinical; prospective randomized double-blind trial	Clinical pRBC units were randomized to prestorage filtration or NLR processing.	194 dogs (96 LR/98 NLR)	pRBC; acute reactions, PCV increment, hospitalization, survival	FNHTR 6.25% LR vs 9.18% NLR (*p* = 0.81); PCV change *p* = 0.08; survival allocation *p* = 0.66
17	Steblaj 2023 [19]	Clinical; retrospective cohort study	Clinically administered pRBC units were categorized as LR or NLR according to blood-bank processing.	339 dogs/413 pRBC units	pRBC; reactions, FNHTR and outcome	TR 19.8% LR vs 17.7% NLR; FNHTR 6.3% vs 4.5%; both *p* > 0.05; no association with discharge
18	Ekiz 2012 [20]	Laboratory; paired controlled 42-day storage study	Each donation produced paired pRBC subunits stored in SAGM; one was filtered before storage and one remained unfiltered.	15 dogs/30 paired pRBC subunits	pRBC in SAGM; hematology, biochemistry and morphology	NLR units had higher leukocyte counts, whereas LR units had higher RBC and hemoglobin values and maintained higher 2,3-DPG concentrations throughout storage; most measured parameters changed over time.
19	McQuinn 2020 [21]	Laboratory; paired split-unit prospective controlled study	RBC units were split into paired subunits; one underwent prestorage filtration and one remained unfiltered.	6 dogs/12 RBC subunits	RBC; NET markers; 42 d	Day-42 cfDNA 0.34 ± 0.82 ng/mL LR vs 1361.07 ± 741.00 NLR (*p* < 0.0001); citrullinated H3 0.19 ± 0.13 vs 0.57 ± 0.34 AU (*p* = 0.007)
20	Miglio 2025 [22]	Laboratory; paired split-unit controlled storage study	pRBC units were divided into paired LR and NLR subunits before storage.	6 dogs/12 pRBC units	pRBC; MDA and free Hb; 42 d	MDA accumulation significantly lower with LR; free Hb lower at day 42 but overall group difference was not significant
21	Miglio 2024 [23]	Laboratory; paired storage/omics study	Each donation produced paired pRBC units prepared with prestorage filtration or without filtration.	8 donors/16 pRBC units	pRBC; metabolomics/lipidomics; 42 d	NLR had more free fatty acids, pyruvate/lactate and amino acids; LR had more ATP, glycolytic and antioxidant metabolites
22	Purcell 2015 [24]	Laboratory; paired split-unit controlled storage study	pRBC units were divided into paired filtered and unfiltered subunits before storage.	12 dogs/24 pRBC subunits	pRBC; IL-8, IL-1β and TNF-α; 37 d	IL-8 higher in NLR at all times and increased after day 10; no LR–NLR difference for TNF-α or IL-1β
23	Shin 2025 [25]	Laboratory; paired split-unit controlled storage study	Each pRBC unit was divided into two aliquots; one was filtered after component preparation and one remained unfiltered.	11 pRBC units/22 aliquots	pRBC; ROS and storage lesions; 42 d	Storage lesions were more severe in NLR; ROS changed in both groups; LR mitigated hemolysis, acidosis and K efflux
24	Hall 2024 [26]	Clinical; prospective multicenter observational surveillance	Clinical transfusions were categorized as LR or NLR according to the processing status of the administered product.	858 dogs/1542 products	Allogeneic products; acute transfusion reactions	Acute TRs occurred in 26/229 (11.4%) LR and 51/635 (8.0%) NLR pRBC transfusions; LR was not associated with lower odds of any TR (OR 1.47, 95% CI 0.89–2.42), FNHTR, AHTR, or allergic reactions
25	Foote 2019 [27]	Laboratory; randomized crossover plasma study	WB was collected with or without prestorage filtration before FFP preparation.	8 dogs/16 FFP units; 14 units analyzed	FFP; coagulation factor activity	No significant LR–NLR differences were detected in coagulation-factor, antithrombin, or protein C activities, PT, aPTT, or fibrinogen; median FVIII and FXI activities exceeded 100% in both groups
26	Spada 2021 [4]	Laboratory; prospective randomized controlled unpaired plasma study	Five randomly selected WB units underwent prestorage filtration and five remained unfiltered before plasma preparation.	10 plasma units (5 LR/5 NLR)	Plasma from prestorage-filtered WB; coagulation activity	Compared with NLR plasma, LR plasma had significantly prolonged aPTT and reduced FXI activity; filtration had no significant effect on the other evaluated hemostatic variables
27	Bala 2017 [28]	Laboratory; controlled cryopreservation study	pRBC samples were assigned to leukocyte filtration or no filtration before cryopreservation at −80 °C.	14 dogs; paired LR and NLR pRBC samples; 12 samples/group reported in the analysis	pRBC; morphology after 4/6 months at −80 °C	Leukocyte filtration did not affect morphological index; cryopreservation and storage increased it
28	Yang 2019 [29]	Laboratory; four-group comparative storage study	WB aliquots were allocated to LR, irradiation, combined LR plus irradiation, or untreated control conditions before storage.	10 dogs/40 WB aliquots allocated to four paired treatment conditions	WB; LR and/or irradiation; cytokines and storage lesions; 28 d	IL-6/CXCL-8 higher in untreated controls; irradiation increased CXCL-8 after day 14 unless blood was LR; storage lesions occurred regardless of LR

Note: AHTR, acute hemolytic transfusion reaction; ATP, adenosine triphosphate; aPTT, activated partial thromboplastin time; CBC, complete blood count; cfDNA, cell-free DNA; CRP, C-reactive protein; EC, erythrocyte concentrate; FFP, fresh-frozen plasma; FNHTR, febrile non-hemolytic transfusion reaction; FXI, coagulation factor XI; Hb, hemoglobin; IL, interleukin; LDH, lactate dehydrogenase; LR, leukoreduced; MCP-1, monocyte chemoattractant protein 1; MDA, malondialdehyde; NLR, non-leukoreduced; NMH, N-methylhistamine; NR, not reported in the full publication; PCV, packed cell volume; PPL, procoagulant phospholipid; pRBC, packed red blood cells; PS, phosphatidylserine; ROS, reactive oxygen species; SAGM, saline-adenine-glucose-mannitol; TR, transfusion reaction; TXB2, thromboxane B2; VEGF, vascular endothelial growth factor; WB, whole blood.

**Table 2:** Predefined criteria used to assess the methodological robustness of the included studies.

Interpretation	Predefined Criteria	Robustness Rating
The study design and reporting provide comparatively reliable support for the reported LR–NLR findings, although the evidence category still determines their clinical directness.	Appropriate direct LR–NLR comparator; randomization or a strong paired/crossover design where applicable; blinding when feasible; adequate sample size; clearly described leukoreduction procedures; predefined and clinically or biologically relevant outcomes; appropriate statistical analysis; complete reporting; and adequate control of relevant confounding factors.	Higher robustness
The findings are informative but should be interpreted in light of identifiable methodological limitations.	Appropriate LR–NLR comparator and an overall valid study design, but with one or more relevant limitations, such as a small sample; absence of blinding; incomplete description of leukoreduction or allocation procedures; multiple exploratory outcomes; limited precision; incomplete sample-size justification; or only partial control of confounding factors.	Moderate robustness
Major methodological limitations substantially reduce confidence in the reported LR–NLR findings.	One or more major limitations, including a very small or incompletely reported sample; weak or unclear allocation methods; substantial risk of selection bias; absence of randomization when applicable; uncontrolled confounding; incomplete outcome reporting; poorly defined outcomes; or insufficiently described or inappropriate statistical analyses.	Lower robustness

Legend: LR, leukoreduced; NLR, non-leukoreduced. Methodological robustness was assessed independently of evidence category and independently of the direction or statistical significance of the study findings. The rating represents an author-defined structured appraisal and not a formally validated risk-of-bias or level-of-evidence scale.

**Table 3:** Evidence directness and methodological robustness of the 28 studies comparing leukoreduced and non-leukoreduced canine blood products.

Main Basis for Classification	Methodological Robustness	Evidence Directness Category	Study
Paired split-unit LR–NLR design reduced donor variability and storage conditions were controlled; however, only seven donors were included and all outcomes were laboratory surrogates.	Moderate	Category D—Product-level evidence	Antognoni 2021 [1]
Randomized controlled storage comparison with a direct LR–NLR comparator; limited by ten units and cytokine measurements without recipient or clinical outcomes.	Moderate	Category D—Product-level evidence	Corsi 2014 [2]
Two-period crossover design with a direct comparison and standardized storage; limited by eight donors and evaluation of a single principal mediator.	Moderate	Category D—Product-level evidence	Frank 2021 [5]
Controlled LR–NLR comparison involving pRBC and plasma components; limited by ten units and VEGF as a surrogate outcome.	Moderate	Category D—Product-level evidence	Graf 2012 [6]
Randomized controlled storage design with quantitative repeated measurements; limited by eleven units and uncertain clinical relevance of microparticle changes.	Moderate	Category D—Product-level evidence	Herring 2013 [7]
A paired split-unit design, repeated sampling, and complementary molecular and culture-based viability assessments were used. Robustness was reduced by the inclusion of only five units, imprecise quantification of the inoculum, possible release of free organisms during mechanical inoculum preparation, and limited statistical handling of the paired repeated observations.	Lower	Category D—Product-level evidence	Lucchese 2020 [8]
Crossover design and standardized storage/simulated-transfusion procedures; limited by eight dogs, multiple biochemical outcomes, and absence of recipient outcomes.	Moderate	Category D—Product-level evidence	Muro 2017 [9]
Randomized controlled storage comparison with an appropriate LR–NLR comparator; limited by ten units and a surrogate procoagulant-phospholipid endpoint.	Moderate	Category D—Product-level evidence	Smith 2015 [10]
Paired split-unit design evaluated multiple blood components and controlled donor variability; limited by small component-specific groups and multiple heterogeneous outcomes.	Moderate	Category D—Product-level evidence	Stefani 2021 [11]
Randomized comparative processing study involving 68 whole-blood collections with clearly defined operational outcomes; limited by insufficient power for some product-weight comparisons and absence of functional or recipient outcomes.	Moderate	Category D—Product-level/operational evidence	Trinder 2022 [12]
Prospective randomized blinded trial in clinically transfused dogs; limited by its pilot design, 23 dogs, and low numbers of transfusion reactions and deaths.	Moderate	Category A—Controlled clinical evidence	Bosch Lozano 2019 [13]
Randomized blinded controlled clinical design with intention-to-treat analysis; limited by a preliminary sample, biomarker-predominant outcomes, and insufficient precision for survival or uncommon clinical events.	Moderate	Category A—Controlled clinical evidence	Claus 2022 [14]
Large two-center clinical cohort including 455 dogs and 730 transfusions; limited by retrospective exposure allocation, possible treatment-selection bias, and residual confounding.	Moderate	Category B—Observational clinical evidence	Davidow 2022 [15]
Randomized prospective autologous transfusion design, crossover comparison of 7- and 28-day storage, repeated recipient measurements, and elimination of compatibility-related confounding; limited by healthy experimental recipients, parallel rather than crossover allocation to LR versus NLR, absence of a reported a priori sample-size calculation for the LR–NLR comparison, and indirect applicability to clinically ill dogs.	Moderate	Category C—Experimental recipient evidence	Callan 2013 [16]
Controlled autologous transfusion study with post-transfusion inflammatory measurements; limited by the small analyzed sample and use of healthy experimental recipients.	Moderate	Category C—Experimental recipient evidence	McMichael 2010 [17]
Prospective randomized double-blind clinical trial with an a priori sample-size calculation, 194 clinically transfused dogs, balanced LR and NLR groups, standardized transfusion monitoring, and relevant clinical outcomes. Limitations included the absence of follow-up for delayed reactions, incomplete control of immunosuppressive treatment and blood-unit age, some missing outcome data, and limited precision for infrequent individual reactions.	Higher	Category A—Controlled clinical evidence	Radulescu 2021 [18]
Large clinical cohort with 339 dogs, 413 pRBC units, and relevant reaction and discharge outcomes; limited by retrospective design, nonrandomized exposure, temporal differences, and residual confounding.	Moderate	Category B—Observational clinical evidence	Steblaj 2023 [19]
Paired split-unit LR–NLR design involving 15 donors, standardized 42-day storage, repeated measurements, and an analysis accounting for storage time and treatment group; limited by the relatively small sample, multiple in vitro outcomes, and absence of recipient data.	Moderate	Category D—Product-level evidence	Ekiz 2012 [20]
Paired split-unit prospective design with a direct LR–NLR comparator and clearly quantified differences; limited by six donors and NET biomarkers without recipient outcomes.	Moderate	Category D—Product-level evidence	McQuinn 2020 [21]
Paired split-unit controlled 42-day storage design with prespecified oxidative and hemolysis markers; limited by six donors and absence of clinical outcomes.	Moderate	Category D—Product-level evidence	Miglio 2025 [22]
Paired LR–NLR design with detailed metabolomic and lipidomic characterization; limited by eight donors, exploratory high-dimensional analyses, and surrogate endpoints.	Moderate	Category D—Product-level evidence	Miglio 2024 [23]
Paired split-unit controlled design with repeated measurements over 37 days; limited by twelve donors and cytokine outcomes measured only in stored products.	Moderate	Category D—Product-level evidence	Purcell 2015 [24]
Paired split-unit LR–NLR design with standardized 42-day storage and repeated measurements; limited by eleven units, multiple laboratory outcomes, and no recipient data.	Moderate	Category D—Product-level evidence	Shin 2025 [25]
Large prospective multicenter study involving 858 dogs and 1542 products, with standardized reaction definitions; limited by nonrandomized LR exposure and possible center-, product-, and patient-related confounding.	Moderate	Category B—Observational clinical evidence	Hall 2024 [26]
Randomized crossover design with a direct LR–NLR comparison and mixed-model analysis; limited by eight dogs, exclusion of two of the 16 FFP units, high variability, low prespecified statistical power, and laboratory hemostatic outcomes without clinical bleeding assessment.	Moderate	Category D—Product-level evidence	Foote 2019 [27]
Prospective controlled comparison with random selection of five units for prestorage LR, standardized processing and storage, and clearly defined hemostatic measurements. Limitations included only five units per group, an unpaired comparison between different donors, use of a single filter type, limited power to detect smaller differences, and absence of recipient or clinical bleeding outcomes.	Moderate	Category D—Product-level evidence	Spada 2021 [4]
A paired LR–NLR comparison was performed after cryopreservation. Robustness was reduced by unexplained inconsistency between the 14 enrolled dogs and the 12 samples per group reported in the analysis, contradictory descriptions of the statistical approach, and reliance on a single morphological outcome.	Lower	Category D—Product-level evidence	Bala 2017 [28]
Four-group controlled design distinguished leukoreduction, irradiation, combined treatment, and untreated control; limited by ten dogs, multiple interventions, and surrogate storage outcomes.	Moderate	Category D—Product-level evidence	Yang 2019 [29]

## Data Availability

No new data were created or analyzed in this study. Data sharing is not applicable to this article.

## Abbreviations

The following abbreviations are used in this manuscript:

CAT	Critically Appraised Topic
IL-8	Interleukin-8
LR	leukoreduced
NLR	non-Leukoreduced
RCT	Randomized Controlled Trial
WB	whole blood
pRBC	packed red blood cell
FFP	fresh-frozen plasma
FNHTR	febrile non-hemolytic transfusion reaction
ROS	reactive oxygen species
VEGF	vascular endothelial growth factors
LDH	Lactate dehydrogenase
NETs	Neutrophil extracellular traps
